# 

**Published:** 1891-10-03

**Authors:** 


					INFANTS
fjHfrajrillWP
"MQMAG'&iii&spr.
.SOatV WES TERN IHFlRMA&t
mwicti hospjjai
WITH EXTRA NURSING SUPPLEMENT.
Saturday, October 3rd, 1891.
ISINGLASS
and INVALIDS.
nnum.

				

